# Race and Ethnicity and Overall Survival in Pediatric Acute Myeloid Leukemia

**DOI:** 10.1001/jamanetworkopen.2026.28602

**Published:** 2026-08-12

**Authors:** Daniel J. Zheng, Long Khuong, Catherine Aftandilian, Kira Bona, Emi H. Caywood, Caitlin W. Elgarten, Brian T. Fisher, Cody-Aaron L. Gathers, Taumoha Ghosh, M. Monica Gramatges, Gary Hettinger, Meret R. Henry, Yuan-Shung V. Huang, Yimei Li, Kelly Maloney, Amir Mian, Tamara P. Miller, Arunkumar Modi, Rajen Mody, Regina M. Myers, Haley Newman, Jose Ortiz, Alix E. Seif, Caroline Smith, Jamie Stokke, Naomi Winick, Jennifer J. Wilkes, Victor Wong, Richard Aplenc, Kelly D. Getz

**Affiliations:** 1 Department of Pediatrics, Perelman School of Medicine, University of Pennsylvania, Philadelphia; 2 Division of Oncology, Children’s Hospital of Philadelphia, Philadelphia, Pennsylvania; 3 Department of Biostatistics, Epidemiology and Informatics, Perelman School of Medicine, University of Pennsylvania, Philadelphia; 4 Department of Pediatrics, Division of Pediatric Hematology, Oncology, Stem Cell Transplantation and Regenerative Medicine, Stanford University, Palo Alto, California; 5 Dana-Farber/Boston Children’s Cancer and Blood Disorders Center, Boston, Massachusetts; 6 Department of Pediatrics, Harvard Medical School, Boston, Massachusetts; 7 Department of Pediatrics, Thomas Jefferson University, Philadelphia, Pennsylvania; 8 Nemours Children’s Health, Wilmington, Delaware; 9 Perelman School of Medicine, University of Pennsylvania, Philadelphia; 10 Division of Infectious Diseases, Children’s Hospital of Philadelphia, Philadelphia, Pennsylvania; 11 Division of Cardiology, Children’s Hospital of Philadelphia, Philadelphia, Pennsylvania; 12 Department of Pediatrics, Division of Pediatric Hematology and Oncology, Primary Children’s Hospital and University of Utah, Salt Lake City; 13 Texas Children’s Hospital, Baylor College of Medicine, Houston, Texas; 14 Department of Population Health, New York University Grossman School of Medicine, New York; 15 Department of Pediatrics, Division of Pediatric Hematology-Oncology and Pediatric Bone Marrow Transplant, Children’s Hospital of Michigan, Detroit; 16 Department of Biomedical and Health Informatics, Children’s Hospital of Philadelphia, Philadelphia, Pennsylvania; 17 Children’s Hospital Colorado, Aurora; 18 Division of Pediatric Hematology/Oncology, Dell Children’s Medical Center, The University of Texas at Austin; 19 Aflac Cancer and Blood Disorders Center, Children’s Healthcare of Atlanta, Atlanta, Georgia; 20 Department of Pediatrics, Emory University School of Medicine, Atlanta, Georgia; 21 Hematology and Oncology, AdventHealth Medical Group Stem Cell Transplant and Cellular Therapy at Orlando, Orlando, Florida; 22 Department of Pediatrics University of Michigan, Ann Arbor; 23 Department of Pediatrics, The University of Texas Southwestern Medical Center, Dallas; 24 Children’s Hospital of Los Angeles, Los Angeles, California; 25 Department of Pediatrics, University of Washington School of Medicine, Seattle; 26 Division of Pediatric Hematology Oncology, Rady Children’s Hospital San Diego, San Diego, California; 27 Department of Pediatrics, University of Colorado School of Medicine, Aurora; 28 Division of Hematology/Oncology, Seattle Children’s Hospital, Seattle, Washington

## Abstract

**Importance:**

Hispanic and non-Hispanic Black (hereafter, Black) children with acute myeloid leukemia (AML) have historically experienced worse survival outcomes compared with non-Hispanic White (hereafter, White) children. Understanding specific intermediate pathways underlying these outcome disparities is a critical step to directing interventional efforts and resources.

**Objective:**

To compare survival outcomes by race and ethnicity in a large contemporary cohort of pediatric patients with AML and to examine presentation acuity, frontline organ toxic effects, and relapse as potential mediators of any observed outcome disparities.

**Design, Setting, and Participants:**

This retrospective cohort study was analyzed from April 2025 to January 2026 and included 13 US children’s hospitals across 10 states participating in the Real-World Epidemiology of Acute Leukemia-AML cohort. Participants included children treated for de novo AML from January 2011 through May 2024.

**Exposure:**

Race and ethnicity (Black, Hispanic, and White).

**Main Outcomes and Measures:**

The main outcome was overall survival (OS), defined as the time from AML diagnosis until death from any cause.

**Results:**

The study cohort included 773 children (median [IQR] age, 9 [2-14] years; 402 males [52.0%]), of whom 125 (16.2%) were Black, 237 (30.7%) were Hispanic, and 411 (53.2%) were White (median [IQR] follow-up, 51.2 [25.7-86.9] months), treated for de novo AML. Among the patients, Black (5-year OS, 61.1% [95% CI, 52.6%-71.0%]; adjusted hazard ratio [AHR], 1.55 [95% CI, 1.26-1.91]) and Hispanic (5-year OS, 65.4% [95% CI, 58.9%-72.5%]; AHR, 1.32 [95% CI, 1.03-1.68]) children had worse OS compared with White children (5-year OS, 70.0% [95% CI, 65.2%-75.1%]). Compared with White patients, Black patients had increased risk of higher cardiovascular (adjusted risk ratio [ARR], 2.09 [95% CI, 1.23-3.56]), respiratory (ARR, 1.36 [95% CI, 1.05-1.77]), and renal (ARR, 3.08 [95% CI, 1.02-9.32]) acuity at presentation. There were no statistically significant increases in severe frontline organ toxic effects or relapse rates by race and ethnicity. Cardiovascular acuity (AHR, 1.83 [95% CI, 1.51-2.22]) and respiratory acuity (AHR, 1.43 [95% CI, 1.15-1.77]) at presentation were independently associated with all-cause mortality. Cardiovascular acuity (proportion mediated, 14%) and respiratory acuity (proportion mediated, 8%) at presentation partially mediated the total absolute survival difference between Black and White children.

**Conclusions and Relevance:**

In this cohort study of pediatric patients with AML, differential cardiovascular and respiratory acuity at presentation were partial mediators of outcome disparities for Black children with AML. Other intermediates along the care continuum should be identified to account for the remainder of the observed outcomes and to prioritize focus areas for interventional efforts to mitigate the survival differences.

## Key Points

**Question:**

Do racial and ethnic differences in presentation acuity, severe frontline organ toxic effects, or relapse rates potentially explain survival disparities in pediatric acute myeloid leukemia (AML)?

**Findings:**

In this cohort study examining 773 pediatric patients with AML treated at 13 US children’s hospitals from 2011 to 2024, there was an overall survival disparity for both Hispanic children and non-Hispanic Black children compared with their non-Hispanic White peers, but they had comparable rates of relapse. Cardiovascular acuity and respiratory acuity at presentation explained up to 14% and 8%, respectively, of the overall survival disparity among Black patients.

**Meaning:**

These findings suggest that overall survival disparities persist for Hispanic and non-Hispanic Black children with AML in the contemporary era, and differential cardiovascular and respiratory acuity at presentation for non-Hispanic Black children is 1 distinct mechanism that may contribute.

## Teaser

This cohort study examines whether racial and ethnic differences in presentation acuity, severe frontline organ toxic effects, or relapse rates are associated with overall survival in pediatric patients with acute myeloid leukemia (AML).

## Introduction

Acute myeloid leukemia (AML) is the second-most-common pediatric hematologic malignant condition, but it is responsible for more than half of the deaths due to pediatric leukemia.^1^ Historically, clinical trials have demonstrated patterns of worse overall survival (OS) for Hispanic and non-Hispanic Black (hereafter, Black) patients compared with non-Hispanic White (hereafter, White) patients.^2,3^ Similar findings for OS have been observed in population-based cohorts and registries.^4,5,6^ Specific pathways through which these outcome disparities arise are unknown. Defining these intermediates is a critical step to focusing interventional efforts and resources.

**Figure 1.**
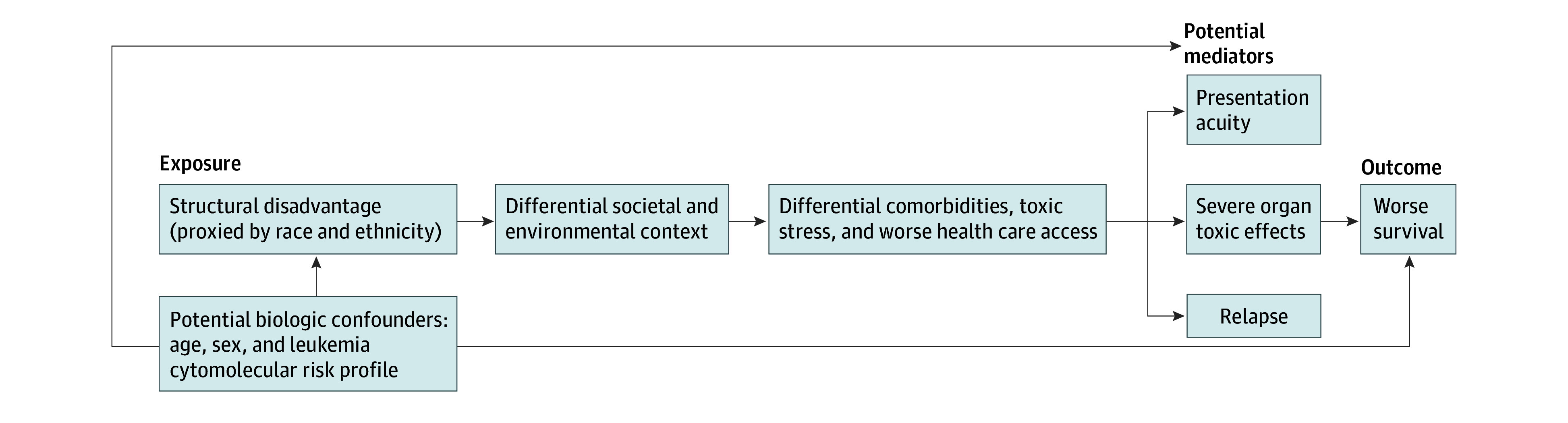
Conceptual Framework A combined race and ethnicity variable was used as a proxy for exposure to structural disadvantage that may predispose individuals to differential societal and environmental contexts, which can lead to differential comorbidities, toxic stress, and worse health care access. Potential downstream mediators and their potential contribution to observed disparities in overall survival were explored.

As a social construct, health disparities by race and ethnicity are posited to reflect the possible downstream consequences of historical and contemporary societal disadvantage (ie, structural racism) for marginalized racial and ethnic groups, which can impact health behavior, health care access, and global stress mechanisms.^7^ In this study, we leveraged race and ethnicity as a proxy variable for exposure to structural racism in a similar conceptual framework to specifically examine potential mediators of survival differences (Figure 1). Prior work in pediatric AML points to 3 potential inflection points along the care continuum that may inform survival disparities by race and ethnicity: presentation acuity, frontline organ toxic effects, and relapse rate. Presentation acuity, defined in prior studies^8,9,10^ as the overall and organ system–specific use of intensive care unit (ICU)–level resources within the first 3 days of initial presentation, has been found to mediate increased induction mortality in Black patients,^8^ but it remains unknown whether this translates into OS disparities, given low rates of induction mortality overall (2%-5%). With regard to frontline organ toxic effects, data from a phase III clinical trial demonstrated greater risk of cardiovascular toxic effects during treatment in Black patients and specifically in the setting of infection.^11^ In terms of relapse, data from the Therapeutically Applicable Research to Generate Effective Treatments (TARGET) clinical trial database demonstrated worse event-free survival for Black patients and Hispanic patients compared with White patients with specific cytomolecular subsets of AML.^12^ However, the TARGET database is subject to selection bias, as it is enriched specifically for patients with relapsed disease and only includes trial-enrolled patients.^13^ Multiple prior studies have demonstrated racial and ethnic disparities in trial enrollment specifically in pediatric AML.^14,15^ In the current study, we used a large, diverse contemporary cohort of pediatric patients with AML to compare OS by race and ethnicity and to evaluate the extent to which outcome disparities may be mediated by 3 potential intermediate pathways: presentation acuity, severe frontline organ toxic effects, and AML relapse.

## Methods

### Data Sources and Collection

This cohort study leveraged data from the Real-World Epidemiology of Acute Leukemia-AML (REAL-AML) cohort, which has been previously described.^15^ REAL-AML includes all pediatric patients diagnosed with AML (aged <19 years at initial diagnosis) from 2011 onward at participating institutions with detailed standardized medical record abstraction. The study protocol was approved by institutional review boards at all participating institutions. Consent requirements were waived for medical record extractions because the research met the criteria of 45 CFR 46.116(f)(3). The majority of REAL-AML institutions also contribute data to the Pediatric Health Information System (PHIS) database. PHIS is a comparative database with daily-level and admission-level clinical and resource utilization data ascertained via emergency department and inpatient billing records from over 40 different children’s hospitals. Our team linked REAL-AML to PHIS data and previously demonstrated a more than 95% success rate in identifying matches when merging with PHIS.^16^ The current study was conducted in accordance with the Strengthening the Reporting of Observational Studies in Epidemiology (STROBE) reporting guideline for cohort studies.

### Study Design and Analytic Cohort

This was a retrospective cohort study that used the February 2025 data freeze of REAL-AML, which included patients diagnosed from January 2011 to May 2024. For this study, we excluded patients who did not receive their first course of induction therapy at the contributing institution to ensure complete capture of acuity at initial presentation and important initial diagnostic information. We also excluded patients with Down syndrome and treatment-related AML, given small numbers and differences in treatment paradigms and overall prognosis, as well as the potential for differential baseline risk for potential mediators of interest. We restricted our study to patients reported in the medical record to be Black, Hispanic, or White. Race and ethnicity were collected from the electronic health record as part of the medical record abstractions conducted for the REAL-AML cohort. In addition, we further restricted our study to the subset of patients for whom we were able to identify a match in PHIS to allow for integration of clinical and resource utilization data necessary to operationalize key variables for the planned analyses. Data were analyzed from April 2025 to January 2026.

### Outcomes and Exposure

The primary outcome was OS, defined as the time from initial AML diagnosis until death from any cause. For this analysis, patients were censored at the date of the last known follow-up or 5 years, whichever occurred sooner. The exposure for this study was a combined race and ethnicity variable operationalized as Black compared with Hispanic compared with White.

### Candidate Mediators

We evaluated 3 prespecified potential mediators for this analysis: presentation acuity (both overall and by specific organ system), severe organ toxic effects during frontline chemotherapy (both overall and by specific organ system), and AML relapse. High-presentation acuity was defined as documented use of organ system-specific ICU-level requirements during the first 3 days of the index hospital admission for an initial AML diagnosis. These organ systems included cardiovascular, respiratory, renal, neurologic, and apheresis, consistent with prior published work (eTable 1 in Supplement 1).^15^ Severe frontline organ toxic effects was defined as use of cardiovascular, respiratory, renal, and neurologic ICU-level resources during frontline AML treatment and was operationalized as a time-to-event outcome. Follow-up for severe frontline organ toxic effects began at the start of induction chemotherapy and continued to the earliest of death, refractory disease, relapse, or completion of frontline therapy. AML relapse was defined as the reappearance of leukemic blasts (≥5%) in the bone marrow, peripheral blood, or extramedullary sites following a prior complete remission. For any analyses involving relapse, we censored patients found to have refractory disease (ie, ≥5% leukemic blasts in the bone marrow or peripheral blood or persistent extramedullary disease at the end of 2 induction cycles).

### Covariates

Baseline covariates from the initial diagnostic period obtained via the medical record included age (continuous and categorical: 0-1.99 years vs 2.00-10.99 years vs ≥11.00 years), sex (male or female), cytomolecular risk (only favorable markers vs only unfavorable markers vs neither vs both), health insurance (any private vs only public vs none vs unknown), and enrollment onto a frontline therapeutic clinical trial (yes or no). The cytomolecular risk profile was defined and operationalized consistent with our team’s prior publication, in which a composite of available karyotype, fluorescence in situ hybridization, and next-generation sequencing–based fusion testing categorized patients into 1 of 4 mutually exclusive groups: only favorable markers, only unfavorable markers, both favorable and unfavorable, and neither.^15^ Individual markers were determined to be favorable or unfavorable based on their designation within the current Children’s Oncology Group clinical trial.^17^ Postinduction measurable residual disease (MRD) was not incorporated into risk classification since this was not known or measurable at baseline and thus could potentially have been on a causal pathway. Within the framework of evaluating race and ethnicity as a proxy variable for exposure to structural and societal disadvantage, we a priori chose not to consider insurance status and enrollment on a frontline clinical trial as potential confounders for the main exposure–outcome association, as these covariates potentially lie on the causal pathway of structural racism’s association with health outcomes via differential health care access (Figure 1).

### Statistical Analysis

Sociodemographic and clinical characteristics were described overall and compared across race and ethnicity groups using χ^2^ and analysis of variance tests as appropriate. Descriptive frequencies were reported for OS and the 3 potential mediators (presentation acuity, severe frontline organ toxic effects, and relapse) overall and by race and ethnicity. Mediation analyses were performed for each candidate mediator separately to decompose the potential total effect of the association of race and ethnicity (exposure) with OS (outcome) into direct and indirect (through the given proposed mediator) pathways. Based on published criteria, decomposition analysis requires (1) a nonnull association between exposure and outcome, (2) a statistically significant association between exposure and the proposed mediator, and (3) a statistically significant association between the proposed mediator and the outcome.^18^ For presentation acuity, organ system–specific variables were analyzed independently rather than within a joint multiple-mediator framework, as they were all ascertained within the same 72-hour presentation window; thus, no clear temporal ordering between them could be established. A 2-sided *P* < .05 was considered statistically significant for all analyses. We assessed the component associations in a stepwise fashion.

First, we assessed the overall exposure–outcome association by using the Kaplan-Meier estimator to calculate OS from the date of initial AML diagnosis and used unadjusted and adjusted Cox proportional hazards regression models with robust SE estimated hazards ratios (HRs) and 95% CIs for the associations between race and ethnicity and OS. Second, we assessed each exposure–potential mediator association. We fit marginal log binomial regression models to estimate risk ratios (RRs) and 95% CIs for the association between race and ethnicity and presentation acuity (overall and by specific organ systems). Parameters were estimated using generalized estimating equations with an exchangeable working correlation structure and robust SEs to account for clustering of observations within centers. Adjusted Cox proportional hazards regression models with robust SEs were used to estimate HRs and 95% CIs for the association between race and ethnicity and severe frontline organ toxic effects (overall and by specific organ systems). We additionally examined the association between race and ethnicity and severe frontline organ toxic effects adjusted for presentation acuity. We plotted the cumulative hazard of relapse with nonrelapse death as a competing risk by race and ethnicity. A cause-specific hazard model estimated the cause-specific HR for relapse. Third, we assessed the potential mediator–outcome association only for mediators that were found to have a statistically significant association with the exposure (ie, race and ethnicity). Cox proportional hazards regression models (with robust SEs), adjusted for age, sex, and cytomolecular risk profile, were used.

For any mediator that met all 3 criteria, we proceeded to decompose the total effect of the association of race and ethnicity with OS into an indirect effect (the pathway through that mediator) and a direct effect (all remaining pathways not through the given mediator).^18^ We calculated the proportion mediated through any specific mediator on the difference scale from the estimates of the direct and indirect effects^18,19^ as



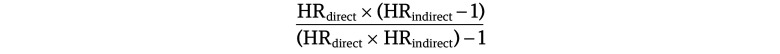


In a sensitivity analysis, we redefined presentation acuity to account for potential misclassification of the potential mediator by using a stricter definition that allowed for less overlap with frontline organ toxic effects. Presentation acuity was redefined as ICU-level requirements from the initial presentation to the start of induction chemotherapy or 72 hours, whichever was earlier. ICU-level requirements within the first 24 hours of presentation were included in presentation acuity regardless of the induction chemotherapy start date.

All analyses were performed using R, version 4.5.1 (R Project for Statistical Computing), with the CMAverse package that provides a suite of functions for reproducible causal mediation analysis.^20^ Additional methodologic details are available in the expanded eMethods in Supplement 1.

## Results

### Study Population

The analytic cohort comprised 773 children diagnosed with AML (median [IQR] age, 9 [2-14] years; 371 female [48.0%] and 402 male [52.0%] patients; 125 Black [16.2%], 237 Hispanic [30.7%], and 411 White [53.2%] patients) at 13 participating US institutions across 10 states from January 2011 through May 2024 (median [IQR] follow-up, 51.2 [25.7-86.9] months) (Table 1 and Table 2; eFigure 1 in Supplement 1). Compared with White patients (126 [30.7%]), Black (83 [66.4%]) and Hispanic (157 [66.2%]) patients at initial AML diagnosis were more likely to be publicly insured (*P* < .001) and less likely to be enrolled in a frontline clinical trial for treatment (Black: 39 [31.2%], Hispanic: 102 [43.8%], and White: 198 [48.4%]; *P* = .003).

**Table 1.** Sociodemographic and Clinical Characteristics of Cohort Overall and by Race and Ethnicity

Characteristic	Participants, No. (%)				*P* value
	Overall (N = 773)	Black (n = 125)	Hispanic (n = 237)	White (n = 411)	
Age at diagnosis, median (IQR), y	9 (2-14)	9 (2-14)	10 (3-14)	9 (2-14)	.35
Age group at diagnosis, y					
0-1.99	171 (22.1)	27 (21.6)	46 (19.4)	98 (23.8)	.55
2.00-10.99	258 (33.4)	47 (37.6)	79 (33.3)	132 (32.1)	
≥11.00	344 (44.5)	51 (40.8)	112 (47.3)	181 (44.0)	
Sex					
Female	371 (48.0)	66 (52.8)	101 (42.6)	204 (49.6)	.11
Male	402 (52.0)	59 (47.2)	136 (57.4)	207 (50.4)	
Cytomolecular risk profile at diagnosis					
Only favorable markers	235 (30.4)	44 (35.2)	81 (34.2)	110 (26.8)	.31
Only unfavorable markers	143 (18.5)	22 (17.6)	44 (18.6)	77 (18.7)	
Neither	388 (50.2)	59 (47.2)	110 (46.4)	219 (53.3)	
Both	7 (0.9)	0	2 (0.8)	5 (1.2)	
Health insurance at diagnosis					
Any private	320 (41.4)	39 (31.2)	53 (22.4)	228 (55.5)	<.001
Public only	366 (47.3)	83 (66.4)	157 (66.2)	126 (30.7)	
None	9 (1.2)	0	4 (1.7)	5 (1.2)	
Unknown	78 (10.1)	3 (2.4)	23 (9.7)	52 (12.6)	
Enrolled on a frontline therapeutic clinical trial	339 (44.2)	39 (31.2)	102 (43.8)	198 (48.4)	.003
Missing, No.	6 (0.8)	0	4 (1.7)	2 (0.5)	

**Table 2.** Clinical Outcomes and Potential Mediators Overall and by Race and Ethnicity

Outcome	Participants, No. (%)			
	Overall (N = 773)	Black (n = 125)	Hispanic (n = 237)	White (n = 411)
5-y Overall survival, % (95% CI)^a^	67.2 (63.6-70.9)	61.1 (52.6-71.0)	65.4 (58.9-72.5)	70.0 (65.2-75.1)
Follow-up among those alive, median (IQR), mo	51.2 (25.7-86.9)	53.9 (29.1-79.1)	50.5 (23.0-81.2)	49.8 (28.3-88.2)
Achieved an initial complete remission	680 (88.0)	103 (82.4)	214 (90.3)	363 (88.3)
**Presentation acuity**				
Cardiovascular	55 (7.1)	15 (12.0)	18 (7.6)	22 (5.4)
Respiratory	240 (31.0)	45 (36.0)	69 (29.1)	126 (30.7)
Renal	19 (2.5)	7 (5.6)	4 (1.7)	8 (1.9)
Neurologic	5 (0.6)	0	2 (0.8)	3 (0.7)
Apheresis	25 (3.2)	3 (2.4)	10 (4.2)	12 (2.9)
Any of the above	253 (32.7)	46 (36.8)	74 (31.2)	133 (32.4)
None of the above	520 (67.3)	79 (63.2)	163 (68.8)	278 (67.6)
**Severe frontline organ toxic effects**				
Cardiovascular	164 (21.2)	32 (25.6)	52 (21.9)	80 (19.5)
Respiratory	408 (52.8)	67 (53.6)	128 (54.0)	213 (51.8)
Renal	51 (6.6)	13 (10.4)	12 (5.1)	26 (6.3)
Neurologic	11 (1.4)	1 (0.8)	4 (1.7)	6 (1.5)
Any of the above (ie, no apheresis)	434 (56.1)	72 (57.6)	139 (58.6)	223 (54.3)
None of the above	339 (43.9)	53 (42.4)	98 (41.4)	188 (45.7)
**Relapse**				
Any	226 (29.2)	37 (29.6)	68 (28.7)	121 (29.4)
Early (≤12 mo)	121 (15.7)	20 (16.0)	36 (15.2)	65 (15.8)
Late (>12 mo)	105 (13.6)	17 (13.6)	32 (13.5)	56 (13.6)
Time to relapse, median (IQR), mo	11.0 (7.22-17.0)	11.3 (6.33-16.7)	11.3 (8.09-16.6)	10.9 (6.52-17.2)

^a^ Estimated using the Kaplan-Meier method.

### Survival by Race and Ethnicity

**Figure 2.**
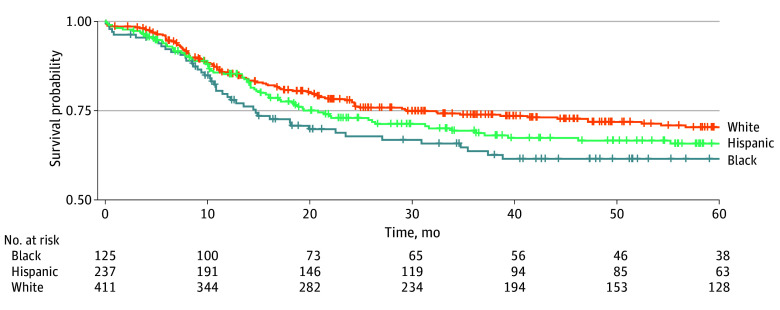
Kaplan-Meier Curve of Overall Survival by Race and Ethnicity Adjusted Cox proportional hazards regression models found increased hazard of death for Hispanic (adjusted hazard ratio, 1.32 [95% CI, 1.03-1.68]; *P* = .03) and Black (adjusted hazard ratio, 1.55 [95% CI, 1.26-1.91]; *P* < .001) patients compared with White patients.

The unadjusted 5-year OS for White patients was 70.0% (95% CI, 65.2%-75.1%) compared with 61.1% (95% CI, 52.6%-71.0%) for Black and 65.4% (95% CI, 58.9%-72.5%) for Hispanic patients (Table 2 and Figure 2). Both Black (adjusted HR [AHR], 1.55 [95% CI, 1.26-1.91]; *P* < .001) and Hispanic (AHR, 1.32 [95% CI, 1.03-1.68]; *P* = .03) patients had an increased hazard of death compared with White patients.

### Presentation Acuity, Severe Frontline Organ Toxic Effects, and Relapse by Race and Ethnicity

Presentation acuity (overall and by organ system) and severe frontline organ toxic effects (overall and by organ system) were largely comparable between Hispanic and White patients. In contrast, Black patients experienced higher acuity at presentation for cardiovascular (adjusted RR [ARR], 2.09 [95% CI, 1.23-3.56]), respiratory (ARR, 1.36 [95% CI, 1.05-1.77]), and renal (ARR, 3.08 [95% CI, 1.02-9.32]) systems compared with White patients (Table 3). Black patients had higher hazards for severe cardiovascular (AHR, 1.40 [95% CI, 0.93-2.09]) and renal (AHR, 1.73 [95% CI, 0.84-3.55]) organ toxic effects during frontline therapy; however, the associations were not statistically significant and were notably attenuated after adjustment for presentation acuity. High cardiovascular (AHR, 1.83 [95% CI, 1.51-2.22]) and respiratory (AHR, 1.43 [95% CI, 1.15-1.77]) acuity at presentation were significantly associated with increased hazards of death, while high renal acuity at presentation was not (eTable 2 in Supplement 1). The hazard for relapse did not differ significantly by race and ethnicity (Table 3; eFigure 2 in Supplement 1). Comparable proportions of patients experienced early relapse by race and ethnicity: 16.0% (20 of 125) for Black, 15.2% (36 of 237) for Hispanic, and 15.8% (65 of 411) for White patients (Table 2).

**Table 3.** Associations Between Race and Ethnicity and Proposed Mediators^**a**^

Potential mediator	Estimate (95% CI)^b^	*P* value
**Presentation acuity, ARR (95% CI)^c^**		
Cardiovascular		
Black	2.09 (1.23-3.56)	.006
Hispanic	1.39 (0.76-2.52)	.28
Respiratory		
Black	1.36 (1.05-1.77)	.02
Hispanic	0.96 (0.74-1.23)	.74
Renal		
Black	3.08 (1.02-9.32)	.05
Hispanic	1.03 (0.33-3.16)	.96
Any system		
Black	1.29 (0.98-1.70)	.07
Hispanic	0.99 (0.76-1.28)	.92
**Severe frontline organ toxic effects, AHR (95% CI)** ^d^ ^,^ ^e^		
Cardiovascular		
Black	1.40 (0.93-2.09)	.11
Hispanic	1.15 (0.86-1.54)	.36
Respiratory		
Black	1.07 (0.70-1.64)	.74
Hispanic	1.06 (0.83-1.36)	.63
Renal		
Black	1.73 (0.84-3.55)	.14
Hispanic	0.83 (0.53-1.30)	.41
Any system (no apheresis)		
Black	1.12 (0.69-1.81)	.65
Hispanic	1.12 (0.89-1.42)	.34
**Severe frontline organ toxic effects controlled for presentation acuity, AHR (95% CI)** ^d^ ^,^ ^e^		
Cardiovascular		
Black	1.06 (0.70-1.61)	.78
Hispanic	1.06 (0.85-1.31)	.62
Respiratory		
Black	1.00 (0.75-1.33)	.98
Hispanic	1.08 (0.94-1.23)	.27
Renal		
Black	1.13 (0.65-1.97)	.67
Hispanic	0.67 (0.50-0.91)	.009
Any system (no apheresis)		
Black	1.07 (0.82-1.40)	.61
Hispanic	1.13 (0.91-1.39)	.28
**Relapse, AHR (95% CI)^f^**		
Black	1.12 (0.90-1.38)	.32
Hispanic	1.07 (0.93-1.26)	.33

Abbreviations: AHR, adjusted hazard ratio; ARR, adjusted risk ratio.

^a^ Reference group for all models is White. Due to the rarity of the events, organ-specific estimates for neurologic and apheresis were not included in the regression analyses.

^b^ All models were adjusted for age, sex, and cytomolecular risk profile.

^c^ Marginal log binomial regression models using generalized estimating equations with robust SEs to account for within-study center correlation.

^d^ Cox proportional hazards regression models with robust SEs accounting for within-study center correlation.

^e^ Operationalized as a time-to-event variable with time defined as the start of induction chemotherapy to the earliest of death, refractory disease, relapse, completion of frontline therapy, or censored at 100 days following hematopoietic cell transplant.

^f^ Cause-specific hazard model.

### Mediation of OS Disparities Through Cardiovascular and Respiratory Acuity at Presentation

Cardiovascular and respiratory acuity at presentation were the only proposed mediators that formally met all predefined criteria to proceed with mediation analysis: (1) nonnull association between exposure and outcome for Black race and OS (AHR, 1.55 [95% CI, 1.26-1.91]; *P* < .001]); (2) statistically significant association between exposure and mediator for Black race and cardiovascular acuity at presentation (ARR, 2.09 [95% CI, 1.23-3.56]; *P* = .006) and for Black race and respiratory acuity at presentation (ARR, 1.36 [95% CI, 1.05-1.77]; *P* = .02); and (3) statistically significant association between mediator and outcome for cardiovascular acuity at presentation and OS (AHR, 1.83 [95% CI, 1.51-2.22]; *P* < .001) and for respiratory acuity at presentation and OS (AHR, 1.43 [95% CI, 1.15-1.77]; *P* = .001). In decomposition analysis, the direct effect of Black race on OS was AHR, 1.46 (95% CI, 0.98-2.14), and the indirect effect mediated through cardiovascular acuity at presentation was AHR, 1.05 (95% CI, 1.00-1.15) (eFigure 3 in Supplement 1). Cardiovascular acuity at presentation mediated a maximum of 14% of the total absolute effect of the association of Black race with OS in this cohort. For respiratory acuity at presentation, the direct effect of Black race on OS was AHR, 1.46 (95% CI, 0.99-2.14), and the indirect effect mediated through respiratory acuity at presentation was AHR, 1.03 (95% CI, 0.99-1.07) (eFigure 3 in Supplement 1). Respiratory acuity at presentation mediated a maximum of 8% of the total absolute effect of the association of Black race with OS in this cohort. Results from the sensitivity analysis using a stricter definition of presentation acuity were similar to findings from the primary analysis (eTable 3 in Supplement 1).

## Discussion

In this contemporary, diverse, cohort study of pediatric patients with AML, we observed a persistent OS disparity for both Black and Hispanic children compared with their White peers despite comparable rates of relapse. Black children experienced over 50%, and Hispanic children experienced over 30% increased hazard of death. The overall survival disparity for Black patients was partially explained by differences in cardiovascular and respiratory acuity at presentation (proportion mediated, 14% and 8%, respectively). Presentation acuity, severe frontline organ toxic effects, and relapse were not identified as potential mediators for outcome differences between Hispanic and White children. Our findings suggest 1 factor that may contribute to survival outcome disparities but highlight the need to identify other intermediates along the care continuum that may account for the remainder of the observed outcomes.

This study explored a widely held but untested assumption that racial and ethnic survival disparities in pediatric AML are mediated by disparities in the rate of relapse. Prior work has focused on clinical trials, which must be interpreted with caution because of observed racial disparities specifically in pediatric AML clinical trial enrollment and because clinical trials have tended to only report event-free survival, which combines induction failure with relapse even though these may represent different clinical entities.^2,12,14^ Conneely et al^12^ published findings using the TARGET database of patients from 3 pediatric AML trials from 1996 to 2013. Similar to findings of older studies from the Children’s Oncology Group,^2^ they found decreased event-free survival for Black patients compared with White patients. However, they did not find any racial and ethnic differences when examining relapse as the first event. Our study identifies a similar finding in an updated contemporary cohort of data as opposed to trial-restricted data.

Our study also builds on prior work from Winestone et al,^8^ who previously reported that acuity of presentation accounted for 61% of the relative excess induction mortality for Black patients. That study was unable to comment on the potential impact on OS, given the specific data source and length of follow-up. Our results suggest that the long-term potential impact, specifically of cardiovascular and respiratory acuity at presentation, is associated with meaningful differences in OS by race and ethnicity. It is unknown whether presentation acuity reflects differences in disease biology, baseline comorbidities, delays in presentation, or some composite. These analyses suggest though that presentation acuity is not primarily explained by disease biology given adjustment for cytomolecular risk profiles, which is also consistent with our team’s prior published work.^15^ As a potential proxy for differential comorbidities and/or delayed presentation, these results may point to the need to examine barriers in health care access. One relevant policy issue to consider is an ability to maintain continuous insurance coverage, with data suggesting that continuous Medicaid coverage is associated with survival benefits in children, adolescents, and young adults with newly diagnosed hematologic malignant conditions as well as lower likelihood of later-stage diagnosis in patients with lymphoma.^21,22^ Importantly, recent estimates from Shen et al^23^ suggest that over 40% of children in the US will experience a period of uninsurance during their childhood, highlighting the magnitude of insurance disruption in a highly fragmented system with complex heterogeneity by state. That study also recapitulated prior findings^23^ that Black and Hispanic children were more likely to be covered under Medicaid. Qualitative data regarding delays in pediatric leukemia diagnosis suggest primary care–focused interventions should also be prioritized, including patient navigation to improve logistical and financial concerns around obtaining timely appointments and practitioner education to mitigate implicit bias that may lead to misattribution of initial symptoms.^24^

### Strengths and Limitations

There are several distinct strengths and innovations of the methods used in this study. This study used data including abstracted information on all patients with newly diagnosed pediatric AML during the study period at participating institutions regardless of trial enrollment status. This provided critical external validity for these results given notable racial disparities specifically in pediatric AML trials.^14^ Importantly, this study also included a diverse cohort with substantive proportions of Black and Hispanic patients. We additionally sought to mitigate measurement error and bias through detailed granular medical record abstraction that was centrally conducted and highly standardized.

There are also some notable limitations. Our primary exposure race and ethnicity variable was collected from electronic medical records, which can have variable practice differences across institutions and time, so there was likely some misclassification. Our definition of relapse was restricted to a historical definition of 5% or more blasts in the marrow and did not account for earlier detection of relapse by MRD, which may differ by race and ethnicity. MRD is a planned future analysis. While we were able to adjust for some biologic confounding using age, sex, and leukemia cytomolecular risk profiles, we did not have data on pharmacogenomics, which may be another mechanism that could contribute to disparities through differential cytarabine metabolism.^25^ In addition, while this was a large multi-institutional cohort spanning 13 institutions across 10 states, it is possible that the sites represented were not representative of a national population.

## Conclusions

In this cohort study of pediatric patients with AML, worse survival outcomes were observed for Black and Hispanic children treated for AML compared with their White peers. These outcomes were notably not explained by differential rates of relapse. Cardiovascular and respiratory acuity at presentation explained a portion of the differential outcomes for Black patients and warrant further study, including their longitudinal association with nonrelapse mortality. Importantly, a large proportion of the observed disparities were not attributable to the examined potential mediators. Ongoing and future work will focus on interrogating other intermediates further downstream in the care continuum, including rates of refractory disease, nonrelapse-related mortality, and survival outcomes after relapse, to identify focus areas of greatest potential impact to mitigate these differences.

## Notes

Supplement 1.
eMethods.
**eTable 1.** Definitions of Organ-Specific ICU Utilization for the Operationalization of Acuity at Presentation and Severe Frontline Organ Toxicity Variables **eFigure 1.** Consort Diagram **eFigure 2.** Cumulative Hazard of Relapse by Race and Ethnicity **eTable 2.** Association Between Cardiovascular, Respiratory, and Renal Presentation Acuity and OS **eFigure 3.** Mediation Analysis Including Estimates of the Direct (Blue) and Indirect (Red) Effects of Non-Hispanic Black Race and Ethnicity With Overall Survival With Cardiovascular Acuity at Presentation (Panel A) and Respiratory Acuity at Presentation (Panel B) as Mediators **eTable 3.** Sensitivity Analysis Leveraging Restricted Definition of Presentation Acuity for Multiple Regression Models for the Association Between Race and Ethnicity and Presentation Acuity and Severe Frontline Organ Toxicity
eReferences.

Supplement 2.
Data Sharing Statement
