# Supplementary material for: Race and Ethnicity and Overall Survival in Pediatric Acute Myeloid Leukemia
Source: JAMA Netw Open. 2026 Aug 12;9(8):e2628602. doi: 10.1001/jamanetworkopen.2026.28602 (PMC13470291; doi:10.1001/jamanetworkopen.2026.28602)
Supplement: Supplementary file 1 [file jamanetwopen-e2628602-s001.pdf]

Supplemental Online Content

Zheng DJ, Khuong L, Aftandilian C, et al. Race and ethnicity and overall survival in pediatric acute myeloid leukemia. *JAMA Netw Open*. 2026;9(8):e2628602. doi:10.1001/jamanetworkopen.2026.28602

eMethods
eTable 1. Definitions of Organ-Specific ICU Utilization for the Operationalization of Acuity at Presentation and Severe Frontline Organ Toxicity Variables
eFigure 1. Consort Diagram
eFigure 2. Cumulative Hazard of Relapse by Race and Ethnicity
eTable 2. Association Between Cardiovascular, Respiratory, and Renal Presentation Acuity and OS
eFigure 3. Mediation Analysis Including Estimates of the Direct (Blue) and Indirect (Red) Effects of Non-Hispanic Black Race and Ethnicity With Overall Survival With Cardiovascular Acuity at Presentation (Panel A) and Respiratory Acuity at Presentation (Panel B) as Mediators
eTable 3. Sensitivity Analysis Leveraging Restricted Definition of Presentation Acuity for Multiple Regression Models for the Association Between Race and Ethnicity and Presentation Acuity and Severe Frontline Organ Toxicity
eReferences

This supplemental material has been provided by the authors to give readers additional information about their work.

© 2026 Zheng DJ et al. *JAMA Network Open*.

eMethods
(expanded Methods section)

Data Sources and Collection
This study leveraged data from the Real-World Epidemiology of Acute Leukemias - AML (REAL-AML) cohort, which has been previously described.[1] REAL-AML includes all pediatric patients diagnosed with AML (age <19 years at initial diagnosis) from 2011 onwards at participating institutions with detailed standardized chart abstraction. The study protocol has been approved by institutional review boards at all participating instructions. Consent requirements were waived for medical record extractions because the research met the criteria of 45 CFR 46.116(f)(3). REAL-AML does not include patients with biphenotypic leukemia, myelodysplastic syndrome without AML transformation, juvenile myelomonocytic leukemia, acute promyelocytic leukemia, or lineage switched AML from a prior diagnosis of acute lymphoblastic leukemia.

Data for REAL-AML is collected via annually updated chart abstraction centrally conducted by researchers at the Children's Hospital of Philadelphia and the University of Pennsylvania. This is part of an ongoing protocol in which incident cases are identified from local cancer registries at each of the participating institutions and prospectively at weekly tumor boards with detailed granular chart abstraction by study personnel. Abstractors are trained using standardized manuals and required to reach >95% concordance with a trainer before commencing study chart reviews. The data at each center is updated at least annually with planned data quality checks and processes for data issue resolution. There are periodic data freezes for interim analyses including this study, which contains data through February 2025.

The majority (N=13) of REAL-AML institutions also contribute data to the Pediatric Health Information System database (PHIS). PHIS is a comparative database with daily- and admission-level clinical and resource utilization data ascertained via emergency department and inpatient billing records from >40 different children's hospitals. We link REAL-AML to PHIS data and previously demonstrated a >95% success rate in identifying matches when merging with PHIS.[2] This research has been conducted in accordance with the Strengthening the Reporting of Observational Studies in Epidemiology (STROBE) reporting guideline for cohort studies.

Study design and analytic cohort
This was a retrospective cohort study that utilized the February 2025 data freeze of REAL-AML which included patients diagnosed from 2011-2024. For this analysis, we excluded patients who did not receive their first course of induction therapy at the contributing institution to ensure complete capture of acuity at initial presentation and important initial diagnostic information. We also excluded patients with Down syndrome and treatment-related AML given small numbers and differences in treatment paradigms and overall prognosis, as well as the potential for differential baseline risk for potential mediators of interest. We restricted our analysis to patients reported in the medical record to be Hispanic, NH-Black, or NH-White. Race and ethnicity were collected from the electronic health record as part of the medical record abstractions conducted for the REAL-AML cohort. Individuals selecting multiple races were classified as Other and ultimately excluded from this analysis. Lastly, we further restricted to the subset of patients for whom we were able to identify a match in PHIS to allow for integration of clinical and resource

© 2026 Zheng DJ et al. *JAMA Network Open*.

utilization data necessary to operationalize key variables for the planned analyses. Data were analyzed from April 2025 to January 2026.

Outcomes

The primary outcome was overall survival (OS), defined as the time from initial AML diagnosis until death from any cause. For this analysis, patients were censored at date of last known follow-up or five years, whichever occurred sooner. Five years was chosen as an administrative censoring cut-off based on the median follow-up time among those who were alive at the time of last follow up to minimize bias from potential differential follow-up time between groups.

Exposure

The exposure for this study was a combined race/ethnicity variable operationalized as Hispanic vs. NH Black vs. NH-White.

Candidate Mediators

We evaluated three pre-specified potential mediators for this analysis: presentation acuity (both overall and by specific organ system), severe organ toxicity during frontline chemotherapy (both overall and by specific organ system), and AML relapse. High presentation acuity was defined as documented utilization of organ system-specific ICU-level requirements during the first 3 days of the index hospital admission for initial AML diagnosis. These organ systems included cardiovascular, respiratory, renal, neurologic, and apheresis, consistent with prior published work (Supplemental Table 1).[1] Severe frontline organ toxicity was defined as utilization of cardiovascular, respiratory, renal, and neurologic ICU-level resources during frontline AML treatment and was operationalized as a time-to-event outcome. Follow-up for severe frontline organ toxicity began at the start of induction chemotherapy and continued to the earliest of death, refractory disease, relapse, or completion of frontline therapy. AML relapse was defined as the reappearance of leukemic blasts (≥5%) in the bone marrow, peripheral blood, or extramedullary sites following a prior complete remission (CR1). For any analyses involving relapse, we censored patients found to have refractory disease (i.e. ≥5% leukemic blasts in the bone marrow or peripheral blood or persistent extramedullary disease at the end of two induction cycles).

Covariates

Baseline covariates from the initial diagnostic period obtained via the medical record included age (continuous and categorical: 0-1.99 years vs. 2-10.99 years vs. ≥11 years), sex (male/female), cytomolecular risk (only favorable markers vs. only unfavorable markers vs. neither vs. both), health insurance (any private vs. only public vs. none vs. unknown), and enrollment onto a frontline therapeutic clinical trial (yes/no). Cytomolecular risk profile was defined and operationalized consistent with our prior publication in which a composite of available karyotype, FISH, and NGS-based fusion testing categorized patients into one of four mutually exclusive groups: only favorable markers, only unfavorable markers, both favorable/unfavorable, and neither.[1] Individual markers were determined to be favorable or unfavorable based on their designation within the current COG AAML1831 clinical trial.[3] Post-induction MRD was not incorporated into risk classification since this is not known/measurable at baseline and thus could potentially be on the casual pathway. Within the framework of evaluating race/ethnicity as a proxy variable for exposure to structural and societal disadvantage, we a priori chose not to consider insurance status and enrollment on a frontline clinical trial as

© 2026 Zheng DJ et al. *JAMA Network Open*.

potential confounders for the main exposure-outcome relationship as these covariates potentially lie on the causal pathway of structural racism's impact on health outcomes via differential healthcare access (Figure 1).

Statistical Analysis
Sociodemographic and clinical characteristics were described overall and compared across race/ethnicity groups using chi-squared and ANOVA tests as appropriate. Descriptive frequencies were reported for OS and the three potential mediators (presentation acuity, severe frontline organ toxicity, and relapse) overall and by race/ethnicity. Mediation analyses were performed for each candidate mediator (presentation acuity, severe frontline organ toxicity, and relapse) separately to decompose the total effect of race/ethnicity (exposure) on OS (outcome) into direct and indirect (through the given proposed mediator) pathways. Based on published criteria, decomposition analysis requires (1) a non-null relationship between exposure and outcome, (2) statistically significant association between exposure and proposed mediator, and (3) statistically significant association between proposed mediator and outcome.[4] For presentation acuity, organ-system-specific variables were analyzed independently rather than within a joint multiple-mediator framework, as they were all ascertained within the same 72-hour presentation window thus no clear temporal ordering between them can be established. A 2-sided $P < .05$ was considered statistically significant for all analyses. We assessed the component associations in a stepwise fashion.

First, we assessed the overall exposure-outcome relationship by utilizing Kaplan-Meier estimator to calculate OS from the date of initial AML diagnosis. Unadjusted and adjusted Cox models with robust standard errors estimated hazards ratios (HR) and 95% confidence intervals (CI) for the associations between race/ethnicity and OS. Second, we assessed each exposure-mediator relationship. We fit marginal log-binomial regression models to estimate risk ratios (RRs) and 95% CIs for the association between race/ethnicity and presentation acuity (overall and by specific organ systems). Parameters were estimated using generalized estimating equations (GEE) with an exchangeable working correlation structure and robust standard errors to account for clustering of observations within centers. Severe frontline organ toxicity was operationalized as a time-to-event variable. Adjusted Cox models with robust standard errors were used to estimate HRs and 95% CIs for the association between race/ethnicity and severe frontline organ toxicity (overall and by specific organ systems). We additionally examined the association between race/ethnicity and severe frontline organ toxicity adjusted for presentation acuity. We plotted the cumulative hazard of relapse with non-relapse death as a competing risk by race/ethnicity. A cause-specific hazard model estimated the cause-specific HR for relapse. Third, we assessed the mediator-outcome relationship only for mediators that demonstrated a statistically significant association with the exposure (i.e. race/ethnicity). Cox models (with robust standard errors) adjusted for age, sex, and cytomolecular risk profile were utilized.

For any mediator that met all three criteria, we proceeded to decompose the total effect of race/ethnicity on OS into an indirect effect (the pathway through that mediator) and a direct effect (all remaining pathways not through the given mediator).[4] We calculated the proportion mediated through any specific mediator on the difference scale from the estimates of the direct and indirect effects as $\frac{HR_{direct} \times (HR_{indirect} - 1)}{(HR_{direct} \times HR_{indirect}) - 1}$.[4, 5]

© 2026 Zheng DJ et al. *JAMA Network Open*.

Sensitivity Analyses

In a sensitivity analysis, we redefined presentation acuity to account for potential misclassification of the mediator by utilizing a stricter definition that allowed for less overlap with frontline organ toxicity. Presentation acuity was redefined as ICU-level requirements from initial presentation to the start of induction chemotherapy or 72 hours, whichever was earlier. ICU-level requirements within the first 24 hours of presentation were included in presentation acuity regardless of induction chemotherapy start date.

All analyses were performed using R version 4.5.1, with the CMAverse package for causal mediation analysis.[6]

© 2026 Zheng DJ et al. *JAMA Network Open*.

eTable 1. Definitions of Organ-Specific ICU Utilization for the Operationalization of Acuity at Presentation and Severe Frontline Organ Toxicity Variables

High presentation acuity was defined as documented utilization of organ system-specific ICU-level requirements during the first 3 days of the index hospital admission for initial AML diagnosis. These organ systems included cardiovascular, respiratory, renal, neurologic, and apheresis. Severe frontline organ toxicity was defined as utilization of cardiovascular, respiratory, renal, and neurologic ICU-level resources during frontline AML treatment and was operationalized as a time-to-event outcome. Follow-up for severe frontline organ toxicity began at the start of induction chemotherapy and continued to the earliest of death, refractory disease, relapse, or completion of frontline therapy.

**System**	**Definition**
Cardiac	This variable was determined at the day-level based on if ANY of the following were true:<br>1. Procedures: ICU-level cardiac care was assigned on a given day if there had been exposure to any of the following procedure codes on that day.<br>• Pulmonary artery wedge monitoring<br>• Systemic arterial pressure monitoring<br>• Continuous intra-arterial blood gas monitoring<br>• Arterial catheterization<br>• Cardiopulmonary resuscitation, not otherwise specified<br>• Other electric countershock of heart<br>• Nonmechanical methods of resuscitation<br>• Measurement of systemic arterial blood gases<br>• Pulmonary artery pressure monitoring<br>2. Medications: ICU-level cardiac care was assigned on a given day if there was exposure to any of the following parenteral medication codes on at least two consecutive days.<br>• Dopamine<br>• Epinephrine<br>• Norepinephrine<br>• Dobutamine<br>• Milrinone<br>• Phenylephrine<br>• Nitroprusside<br>• Amiodarone<br>• Esmolol<br>• Isoproterenol<br>3. Clinical Resources: ICU-level cardiac care was assigned on a given day if there was exposure to any of the following clinical resources that day.

	• Arterial catheter insertion<br>• Cardiopulmonary resuscitation<br>• Cardioversion/defibrillation<br>• Arterial line monitoring<br>• Pulmonary artery line monitoring
Respiratory	This variable was determined at the day-level based on if ANY of the following were true:<br>1. Procedures: ICU-level respiratory care was assigned on a given day if there had been exposure to any of the following procedure codes on that day.<br>• Ventilator management<br>• ECMO<br>2. Medications: ICU-level respiratory care was assigned on a given day if there was exposure to any of the following parenteral medication codes on at least two consecutive days.<br>• Aminophylline<br>• Theophylline<br>3. Clinical Resources: ICU-level respiratory care was assigned on a given day if there was exposure to any of the following clinical resources that day.<br>• Ventilation assistance, unspecified<br>• High frequency ventilation<br>• CPAP<br>• BiPAP<br>• Noninvasive positive pressure ventilatory support<br>• Mechanical ventilation<br>• Manual ventilation<br>• Other specified vent assistance<br>• Oxygen therapy<br>• ECMO<br>• Nitric Oxide<br>• High flow nasal cannula<br>• Surfactant therapy
Renal	This variable was determined at the day-level based on if ANY of the following were true:<br>1. Procedures: ICU-level renal care was assigned on a given day if there had been exposure to any of the following procedure codes on that day.<br>• Venous catheterization for renal dialysis<br>• Hemodialysis<br>• Peritoneal dialysis<br>• Arteriovenostomy for renal dialysis

© 2026 Zheng DJ et al. *JAMA Network Open*.

	2. Medications: ICU-level renal care was assigned on a given day if there was exposure to any of the following parenteral medication codes on at least two consecutive days.<br>• Dialysis solutions, unspecified<br>• Other specified dialysis solutions<br>• Dextrose 1.5% base solution<br>• Dextrose 2.5% base solution<br>• Dextrose 4.25% base solution<br>3. Clinical Resources: ICU-level renal care was assigned on a given day if there was exposure to any of the following clinical resources that day.<br>• Hemodialysis<br>• Peritoneal dialysis<br>• CAVH
Neurologic	This variable was determined at the day-level based on if ANY of the following were true:<br>1. Procedures: ICU-level neurologic care was assigned on a given day if there had been exposure to any of the following procedure codes on that day.<br>• Intracranial pressure monitoring<br>2. Medications: ICU-level neurologic care was assigned on a given day if there was exposure to any of the following parenteral medication codes on at least two consecutive days.<br>• Mannitol<br>3. Clinical Resources: ICU-level neurologic care was assigned on a given day if there was exposure to any of the following clinical resources that day.<br>• Intracranial pressure monitoring
Hematologic/Apheresis	This variable was determined at the day-level based on if ANY of the following were true:<br>1. Procedures: ICU-level hematologic/apheresis care was assigned on a given day if there had been exposure to any of the following procedure codes on that day.<br>• Therapeutic leukopheresis<br>• Plasmapheresis<br>• Pheresis of plasma, multiple

© 2026 Zheng DJ et al. *JAMA Network Open*.

eFigure 1. Consort Diagram

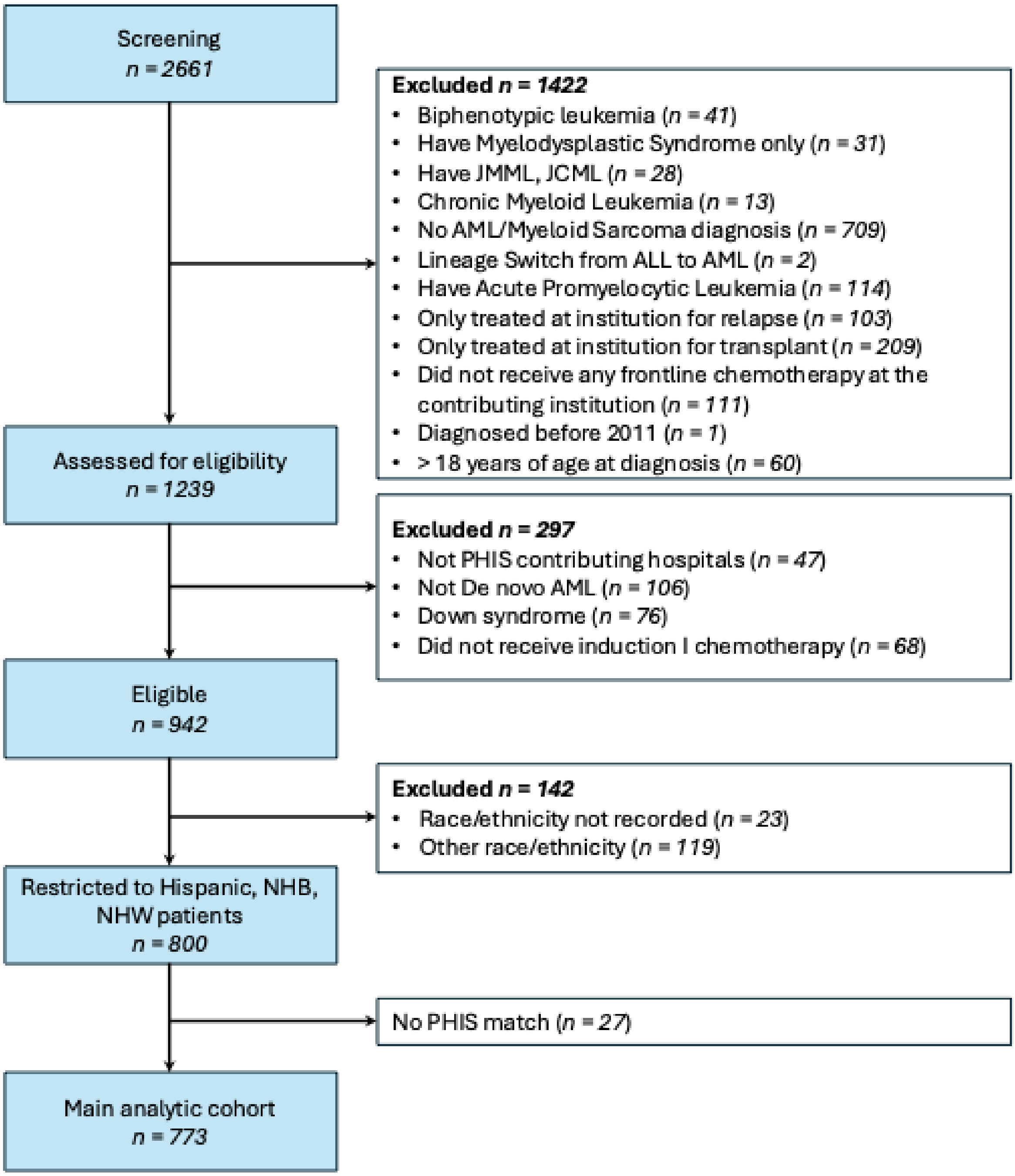

© 2026 Zheng DJ et al. *JAMA Network Open*.

eFigure 2. Cumulative Hazard of Relapse by Race and Ethnicity

The adjusted cause-specific hazard for relapse did not differ significantly for Hispanic (AHR, 1.07; 95% CI, 0.93 – 1.26; p=.33) and NH-Black (AHR, 1.12; 95% CI, 0.90 – 1.38; p=.32) patients compared to NH-White patients.

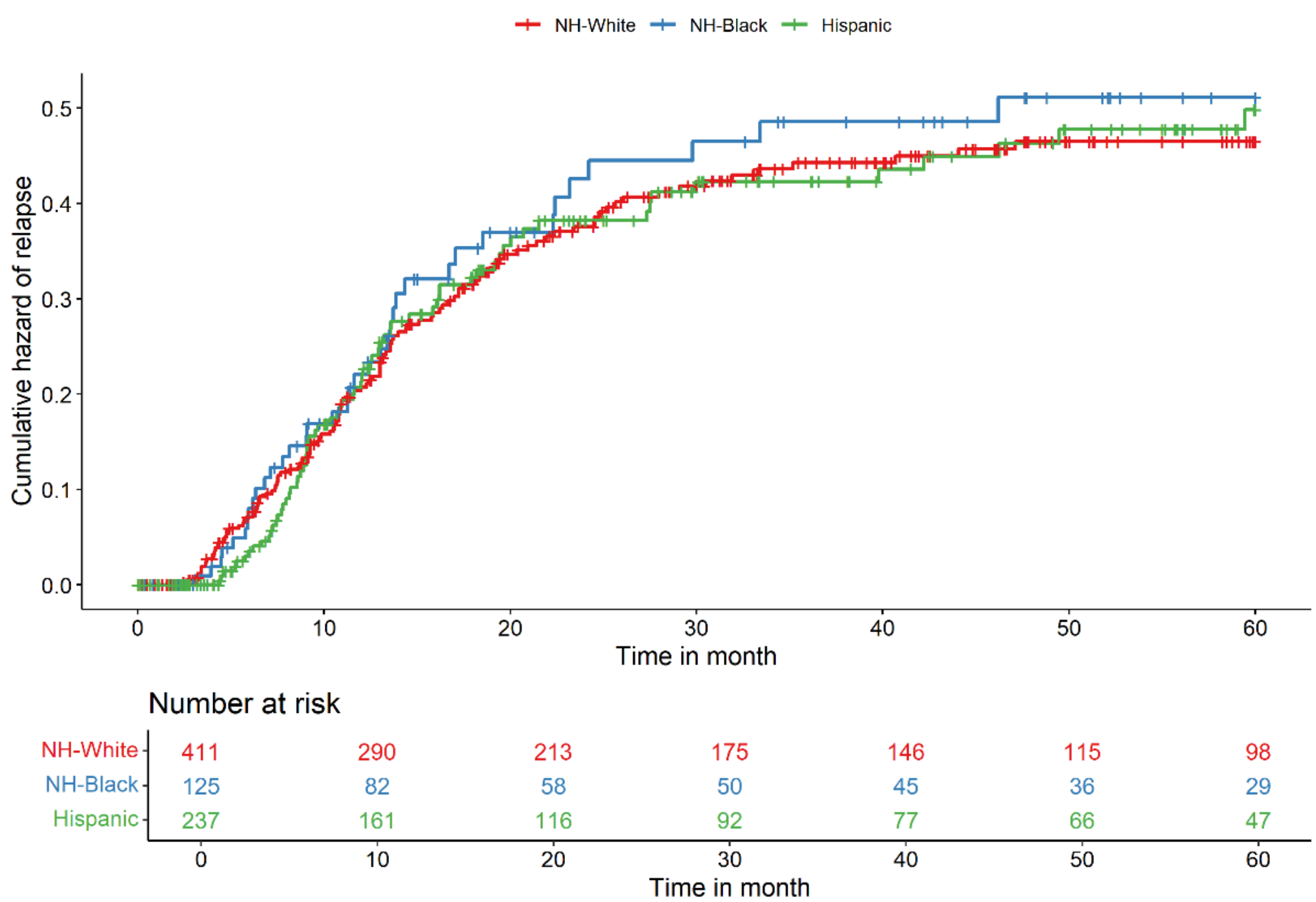

© 2026 Zheng DJ et al. *JAMA Network Open*.

eTable 2. Association Between Cardiovascular, Respiratory, and Renal Presentation Acuity and OS

	Overall survival	
	**AHR (95%CI)**	**p-value**
**Presentation Acuity[a]**		
**Cardiovascular**, *(Ref: No)*		
Yes	1.83 (1.51 – 2.22)	<.001
**Respiratory**, *(Ref: No)*		
Yes	1.43 (1.15 – 1.77)	.001
**Renal**, *(Ref: No)*		
Yes	1.30 (0.70 – 2.42)	.41

[a]Cox PH models with robust standard errors accounting for within study center correlation. All models are adjusted for age, sex, and cytomolecular risk profile.

© 2026 Zheng DJ et al. *JAMA Network Open*.

eFigure 3. Mediation Analysis Including Estimates of the Direct (Blue) and Indirect (Red) Effects of Non-Hispanic Black Race and Ethnicity With Overall Survival With Cardiovascular Acuity at Presentation (Panel A) and Respiratory Acuity at Presentation (Panel B) as Mediators.

A.

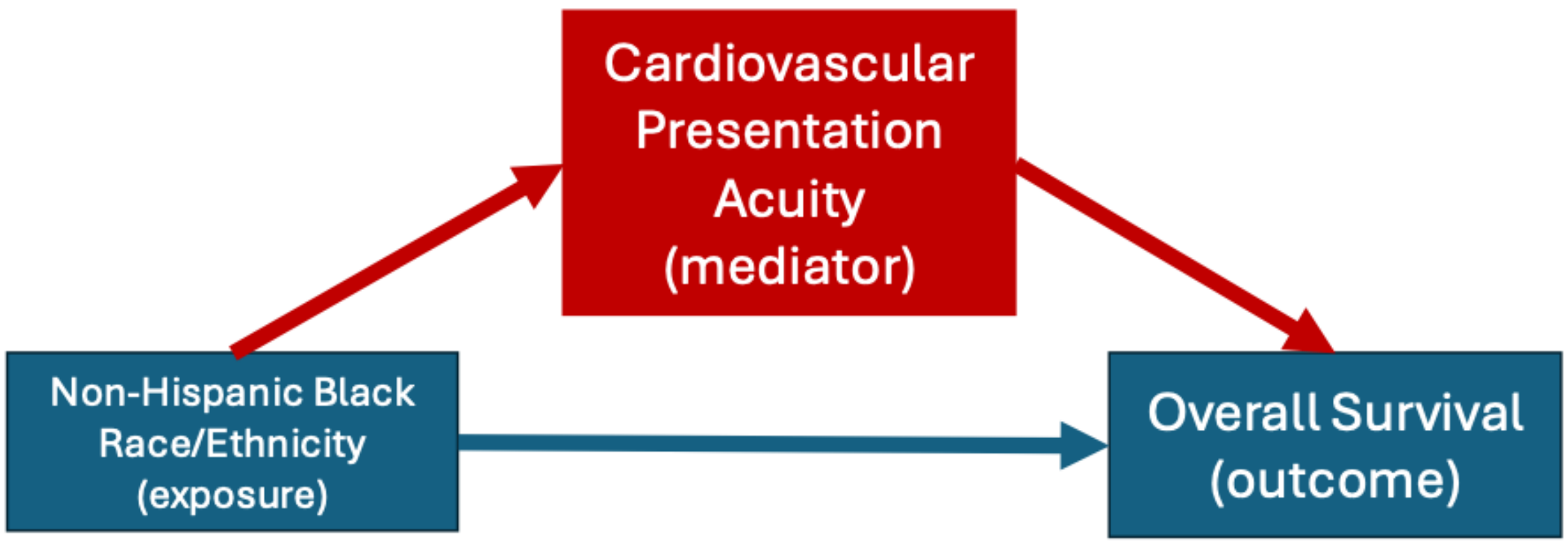

Total Effect of Black Race on OS, AHR 1.53 (1.05-2.22)
→ **Indirect Effect** of Black Race Through Cardiovascular Presentation Acuity, AHR 1.05 (1.00-1.15)
→ **Direct Effect** of Black Race Through Other Pathways, AHR 1.46 (0.98-2.14)

B.

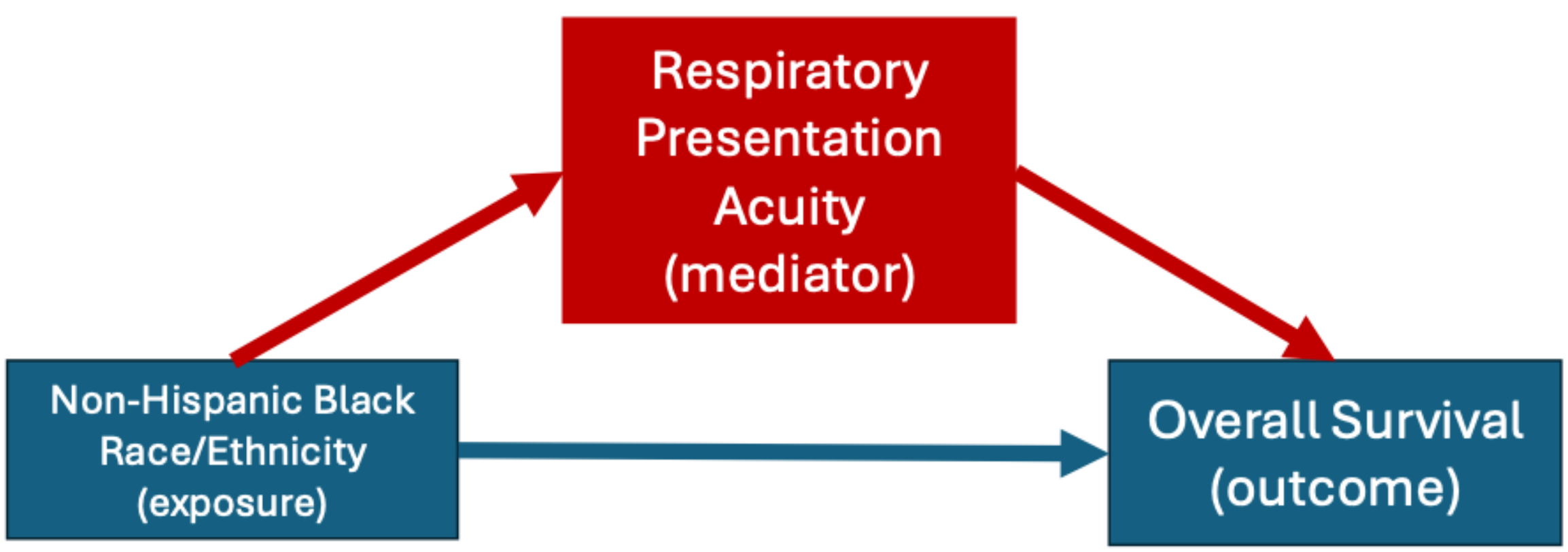

Total Effect of Black Race on OS, AHR 1.53 (1.05-2.22)
→ **Indirect Effect** of Black Race Through Respiratory Presentation Acuity, AHR 1.03 (0.99-1.07)
→ **Direct Effect** of Black Race Through Other Pathways, AHR 1.46 (0.99-2.14)

© 2026 Zheng DJ et al. *JAMA Network Open*.

eTable 3. Sensitivity Analysis Leveraging Restricted Definition of Presentation Acuity for Multiple Regression Models for the Association Between Race and Ethnicity and Presentation Acuity and Severe Frontline Organ Toxicity

In this analysis, presentation acuity was redefined as ICU-level requirements from initial presentation to the start of induction chemotherapy or 72 hours, whichever was earlier. ICU-level requirements within the first 24 hours of presentation were included in presentation acuity regardless of induction chemotherapy start date.

	**Estimate (95%CI)**	**p-value**
**Presentation acuity[§]**	**ARR (95%CI)**	
**Cardiovascular**		
Hispanic	1.68 (0.73 – 3.84)	.22
NH-Black	2.24 (0.94 – 5.38)	.07
**Respiratory**		
Hispanic	1.08 (0.83 – 1.41)	.57
NH-Black	1.49 (1.01 – 2.20)	.05
**Renal**		
Hispanic	2.02 (0.40 – 10.20)	.40
NH-Black	3.40 (0.79 – 14.70)	.10
**Any system**		
Hispanic	1.16 (0.88 – 1.52)	.29
NH-Black	1.48 (1.00 – 2.18)	.05
**Time to severe frontline organ toxicity[¶]**	**AHR (95% CI)**	
**Cardiovascular**		
Hispanic	1.15 (0.86 – 1.54)	.36
NH-Black	1.40 (0.93 – 2.09)	.11
**Respiratory**		
Hispanic	1.06 (0.83 – 1.36)	.63
NH-Black	1.07 (0.70 – 1.64)	.74
**Renal**		
Hispanic	0.83 (0.53 – 1.30)	.41
NH-Black	1.73 (0.84 – 3.55)	.14
**Any system (**no apheresis)		
Hispanic	1.12 (0.89 – 1.42)	.34
NH-Black	1.12 (0.69 – 1.81)	.65
**Time to severe frontline organ toxicity controlled for presentation acuity[¶,]**	**AHR (95% CI)**	
**Cardiovascular**		
Hispanic	1.11 (0.85 – 1.45)	.46
NH-Black	1.29 (0.87 – 1.90)	.20
**Respiratory**		
Hispanic	1.03 (0.86 – 1.24)	.72
NH-Black	1.02 (0.73 – 1.44)	.89
**Renal**		

© 2026 Zheng DJ et al. *JAMA Network Open*.

Hispanic	0.69 (0.48 – 1.00)	.05
NH-Black	1.45 (0.76 – 2.76)	.26
**Any system (**no apheresis)		
Hispanic	1.08 (0.89 – 1.31)	.46
NH-Black	1.08 (0.72 – 1.61)	.71

[§] GEE models with robust standard errors accounting for within study center correlation, [¶]Cox models with robust standard errors accounting for within study center correlation. All models are adjusted for age, sex, and cytomolecular risk profile. Reference group for all models is NH-White. Due to rarity of events, organ-specific ICU utilization for neurologic and apheresis conditions are not included in the regression analysis.

© 2026 Zheng DJ et al. *JAMA Network Open*.

eReferences

1. Zheng DJ, Hettinger G, Aftandilian C, et al. Real-world generalizability of clinical trial cytomolecular risk in pediatric acute myeloid leukemia: a report from the REAL-AML cohort. *JNCI: Journal of the National Cancer Institute*. 2025:djaf234.
2. Getz KD, Szymczak JE, Li Y, et al. Medical outcomes, quality of life, and family perceptions for outpatient vs inpatient neutropenia management after chemotherapy for pediatric acute myeloid leukemia. *JAMA network open*. 2021;4(10):e2128385-e2128385.
3. Cooper TM, Ries RE, Alonzo TA, et al. Revised risk stratification criteria for children with newly diagnosed acute myeloid leukemia: a report from the Children's Oncology Group. *Blood*. 2017;130(Supplement 1):407-407.
4. VanderWeele T. *Explanation in causal inference: methods for mediation and interaction*. Oxford University Press; 2015.
5. Valeri L, VanderWeele TJ. Mediation analysis allowing for exposure–mediator interactions and causal interpretation: theoretical assumptions and implementation with SAS and SPSS macros. *Psychological methods*. 2013;18(2):137.
6. Shi B, Choirat C, Coull BA, VanderWeele TJ, Valeri L. CMAverse: a suite of functions for reproducible causal mediation analyses. *Epidemiology*. 2021;32(5):e20-e22.

© 2026 Zheng DJ et al. *JAMA Network Open*.
