# Supplementary material for: Race and Ethnicity and Overall Survival in Pediatric Acute Myeloid Leukemia
Source: JAMA Netw Open. 2026 Aug 12;9(8):e2628602. doi: 10.1001/jamanetworkopen.2026.28602 (PMC13470291; doi:10.1001/jamanetworkopen.2026.28602)
Supplement: Supplementary file 2 [file jamanetwopen-e2628602-s002.pdf]

Data Sharing Statement

Zheng. Race and Ethnicity and Overall Survival in Pediatric Acute Myeloid Leukemia. *JAMA Netw Open*. Published August 12, 2026. doi:10.1001/jamanetworkopen.2026.28602

Data
Data available: Yes
Data types: Deidentified participant data
How to access data: Deidentified data may be made available with appropriate justification upon request to the cohort investigators and establishment of a Data Use Agreement.
When available: With publication

Supporting Documents
Document types: None

Additional Information
Who can access the data: Deidentified data may be made available with appropriate justification upon request to the cohort investigators and establishment of a Data Use Agreement.
Types of analyses: Deidentified data may be made available with appropriate justification upon request to the cohort investigators and establishment of a Data Use Agreement.
Mechanisms of data availability: Deidentified data may be made available with appropriate justification upon request to the cohort investigators and establishment of a Data Use Agreement.
